# The use of alteplase, although safe, does not offer clear clinical advantages when mild stroke is non-disabling

**DOI:** 10.3389/fneur.2023.1212712

**Published:** 2023-07-17

**Authors:** Giovanni Merlino, Lorenzo Nesi, Pietro Vergobbi, Marco Domenico Scanni, Sara Pez, Alessandro Marziali, Yan Tereshko, Giuseppe Sportelli, Simone Lorenzut, Francesco Janes, Gian Luigi Gigli, Mariarosaria Valente

**Affiliations:** ^1^Stroke Unit, Department of Head-Neck and Neuroscience, Udine University Hospital, Udine, Italy; ^2^Clinical Neurology, Udine University Hospital, Udine, Italy; ^3^Dipartimento di Area Medica (DAME), University of Udine, Udine, Italy

**Keywords:** mild stroke, non-disabling stroke, alteplase, intravenous thrombolysis, NIHSS

## Abstract

**Introduction:**

It is unknown whether alteplase is effective and safe in patients with mild acute ischemic stroke (AIS). Determining whether symptoms are “disabling” or not is a crucial factor in the management of these patients. This study aimed to investigate the efficacy and safety of alteplase in patients with mild, non-disabling AIS.

**Methods:**

We included all consecutive patients admitted for AIS at our institution from January 2015 to May 2022 who presented a baseline NIHSS score of 0–5 and fit the criteria to receive intravenous thrombolysis. In order to select only subjects with non-disabling AIS, we excluded patients who scored more than 1 point in the following NIHSS single items: vision, language, neglect, and single limb. Patients who scored at least 1 point in the NIHSS consciousness item were excluded as well. This study is a retrospective analysis of a prospectively collected database.

**Results:**

After the application of the exclusion criteria, we included 319 patients, stratified into patients receiving and not receiving alteplase based on non-disabling symptoms. The two groups were comparable regarding demographic and clinical data. Rates of a 3-month favorable outcome, defined as a 3-month mRS score of 0–1, were similar, being 82.3% and 86.1% in the treated and untreated patients, respectively. Hemorrhagic complications and mortality occurred infrequently and were not affected by alteplase treatment.

**Discussion:**

This observational study suggests that the use of alteplase, although safe, is not associated with a better outcome in highly selected patients with non-disabling AIS.

## Introduction

The prevalence of patients affected by mild acute ischemic stroke (AIS) is as high as 50%, and a large proportio*n* (37%) of these patients will be disabled 90 days after stroke (1, 2). Thus, treatment of mild AIS remains a challenge for vascular neurologists.

Although several observational studies faced this problematic topic, they adopted dissimilar definitions of mild AIS and achieved conflicting results (2–9). The recent randomized PRISMS study did not prove functional outcome benefits in patients with mild stroke treated with alteplase as compared to aspirin therapy. On the contrary, the rate of symptomatic intracerebral hemorrhage (SICH) was increased in the alteplase group. However, the trial's early termination precluded any definitive conclusions on this topic (10).

As underlined by a recent review, determining whether symptoms are “disabling” or not is a crucial factor in the management of acute mild stroke (11). Using the Barthel index (BI) for measuring disability, we demonstrated that AIS patients with a National Institutes of Health Stroke Scale (NIHSS) score of ≤5 responded differently to alteplase based on their level of functional dependence (12). Unlike the NIHSS, which is routinely utilized by vascular neurologists, the BI is rarely used by physicians (13). Thus, we are aware that our previous results, albeit interesting, are difficult to apply in everyday clinical practice.

The ARAMIS trial has been designed to explore the efficacy and safety of dual antiplatelet therapy vs. alteplase in Chinese patients with mild AIS. Awaiting the results of this trial, we decided to analyze our database of consecutive patients with AIS, adopting the ARAMIS definition of non-disabling stroke, which is strictly based on the NIHSS (14). Thus, this observational, retrospective study aimed to investigate the efficacy and safety of intravenous thrombolysis in highly selected subjects with non-disabling AIS.

## Methods

### Patients

This study included all consecutive patients admitted at our institution for AIS from January 2015 to May 2022 who presented with a baseline NIHSS score of 0–5. Patients were excluded if (1) they had an acute stroke due to large vessel occlusion; (2) the delay from the onset of symptoms to treatment was more than 4.5 h; (3) their premorbid modified Rankin Scale (mRS) was more than 1; and 4) they had absolute contraindications to alteplase, such as the presence of a therapeutic level of anticoagulation at admission. In addition, in order to select only subjects with non-disabling AIS, we excluded patients who scored more than 1 point in the following NIHSS single items: vision, language, neglect, and single limb. Patients who scored at least 1 point in the NIHSS consciousness item were also excluded. Adopting the above-reported exclusion criteria, we are confident that included patients with non-disabling deficits were treated or not treated with alteplase based only on how clinical symptoms were perceived by the vascular neurologist.

Alteplase was administered at a dosage of 0.9 mg/kg (10% bolus and 90% as a 1-h infusion) with a maximum dose of 90 mg. According to the previous versions of the American guidelines (15, 16), we did not obtain any informed consent from our non-disabled patients admitted before 2019. From 2019 onwards, when the current American guidelines were published, we obtained written informed consent from all patients with mild, non-disabling stroke symptoms who were treated with alteplase (17). Patients who were not treated with alteplase received antiplatelets or anticoagulants based on their stroke etiology. In patients presenting with minor non-cardioembolic ischemic stroke, we used aspirin or clopidogrel monotherapy, a loading dose (aspirin 300 mg or clopidogrel 300 mg) within the first 24 h after the onset, followed by a maintenance dose (aspirin 100 mg or clopidogrel 75 mg). In 2019, the American guidelines strongly supported treatment with dual antiplatelet therapy (DAPT), aspirin, and clopidogrel in patients affected by minor non-cardioembolic AIS (17). From that moment on, this specific pharmacological approach was adopted at our institution. In particular, patients started DAPT within 24 h from symptom onset using a loading dose of 300 mg aspirin and 300 mg clopidogrel, followed by 100 mg aspirin and 75 mg clopidogrel from the second day until the third week. Patients with cardioembolic stroke were treated with anticoagulant therapy. Patients started anticoagulants based on the “1-3-6-12 days rule” that was introduced in 2013 by the European Heart Rhythm Association of the European Society of Cardiology (18).

This study conformed to the Declaration of Helsinki of the World Medical Association and was approved by the local ethics committee (Ref. No. CEUR-2020-Os-173).

### Data collection

We collected the following information: age, sex, vascular risk factors (previous transient ischemic attack or stroke, cardiovascular disease, atrial fibrillation, hypertension, diabetes mellitus, hypercholesterolemia, and active tobacco use), laboratory findings, admission systolic and diastolic blood pressure, and antithrombotic therapy before and after the AIS.

Information on extracranial vessel status was obtained using ultrasound echo-color Doppler or CT angiography. The NASCET criteria were used for grading stenosis (19). The trial of ORG 10,172 in acute stroke treatment (TOAST) criteria were used for classifying AIS into different etiologies (20). Stroke severity was quantified at admission and discharge using the NIHSS score. The degree of previous functional disability was calculated at admission based on pre-stroke disability and 3 months after stroke using the mRS. The mRS score after discharge was recorded during the patient's routine clinical visits or through telephone interviews with patients or their immediate caregivers. The European Cooperative Acute Stroke Study (ECASS) definition of parenchymal hematoma types 1 and 2 was adopted to identify intracranial hemorrhage (ICH) (21). Based on the ECASS III protocol, the presence of symptomatic intracranial hemorrhage (SICH) was defined as any hemorrhage with neurological deterioration, as indicated by an NIHSS score that was higher by ≥4 points than the value at the baseline, the lowest value in the first 7 days, or any hemorrhage leading to death (22).

### Outcome measures

The following endpoints were analyzed: (1) 3-month favorable outcome, defined as a 3-month mRS score of 0–1; (2) 3-month functional independence, defined as a 3-month mRS score of 0–2; (3) relevant neurological deterioration at discharge, defined as an impairment of >4 points on the NIHSS score from baseline or leading to death; (4) in-hospital mortality; (5) 3-month mortality; (6) the presence of ICH; and (7) the presence of SICH.

Physicians who assessed the outcome measures were unblinded regarding the patients who received acute stroke therapy.

### Statistical analysis

Data are displayed in tables as the mean and standard deviation unless otherwise specified. The Kolmogorov–Smirnov test with Lilliefor's significant correction was performed to test the normality of the variables. Statistical comparisons were performed using the chi-square test or Fisher's exact test, when appropriate, for categorical variables. Differences between the two groups were assessed using the Student's *t*-test for the independent sample when variables had a normal distribution and by the Mann–Whitney U test when variables had an abnormal distribution. Multivariate logistic regression models were used to evaluate the association of using alteplase with the endpoints. These models were adjusted for age, sex, and baseline NIHSS score. All probability values were two-tailed. Statistical significance was set at *p* < 0.05. Statistical analysis was carried out using IBM SPSS Statistics for Windows, version 26.0 (IBM Corp., Armonk, NY, USA).

## Results

### General and clinical characteristics

**Figure 1:**
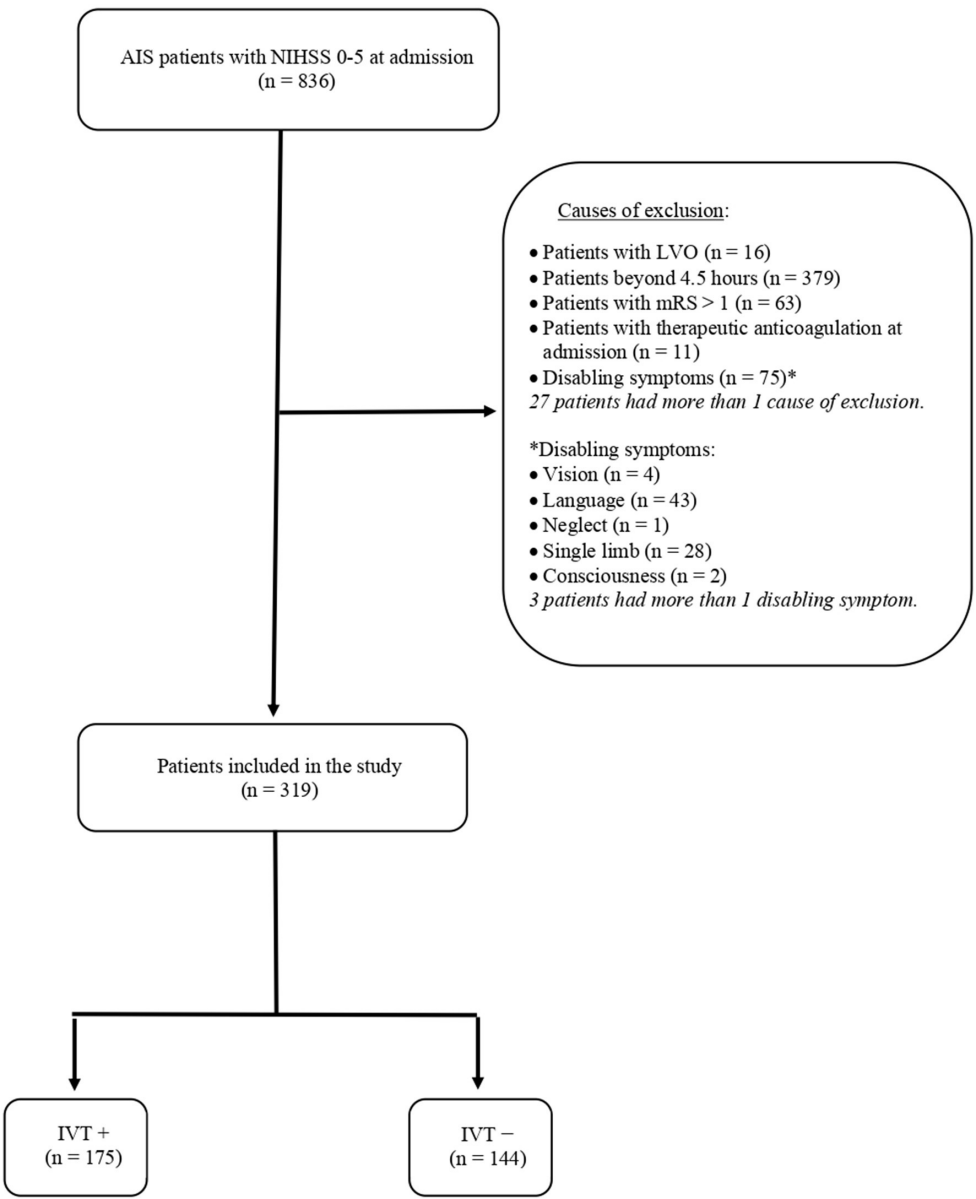
Flow diagram of the study. AIS, acute ischemic stroke; NIHSS, National Institutes of Health Stroke Scale; LVO, large vessel occlusion; mRS, modified Rankin Scale; IVT, intravenous thrombolysis.

During the study period, 836 patients were admitted for AIS with an NIHSS score of 0–5. Of them, 544 patients were excluded due to prespecified exclusion criteria, which are summarized in Figure 1. The remaining 319 AIS patients were included in the study and distinguished into subjects who received intravenous thrombolysis (IVT+) and subjects to whom alteplase was denied because of non-disabling symptoms (IVT–). The flow diagram of the study is reported in Figure 1.

As reported in Table 1, the two groups were comparable regarding demographic and clinical data. Indeed age, sex, and vascular risk factors did not differ between subjects receiving and not receiving alteplase. Similarly, causes of ischemic stroke were superimposable between IVT+ and IVT–patients. Regarding pre-stroke antithrombotic treatment, the use of antiplatelets was not different between the two groups, whereas 17 patients used anticoagulants prior to the index event. Of these, 11 were excluded because of therapeutic levels of anticoagulation at admissio*n* (see Figure 1), whereas 6, having subtherapeutic anticoagulation, were treated with alteplase. Post-stroke use of antiplatelets or anticoagulants did not differ between IVT+ and IVT– patients, although rates of DAPT were significantly higher in subjects not treated with alteplase. The only significant differences between the two groups were lower levels of aPTT and higher levels of fasting glucose in the group of subjects treated with intravenous thrombolysis. In addition, as expected, strokes were significantly more severe in the IVT+ group.

**Table 1:** General characteristics according to treatment with intravenous thrombolysis.

	**IVT + (*n* = 175)**	**IVT – (*n* = 144)**	***p***
**Demographic data**			
Age, years^*^	71 (60-79)	70 (59-78)	0.276
Male sex, *n* (%)	111 (63.4)	99 (68.8)	0.319
**Vascular risk factors**			
Previous transient ischemic attack/stroke, *n* (%)	10 (5.7)	14 (9.7)	0.177
Cardiovascular disease, *n* (%)	21 (12.1)	21 (14.6)	0.510
Atrial fibrillation, *n* (%)	15 (8.6)	10 (6.9)	0.591
Hypertension, *n* (%)	108 (61.7)	90 (62.5)	0.886
Diabetes mellitus, *n* (%)	26 (14.9)	24 (16.7)	0.658
Hypercholesterolemia, *n* (%)	54 (30.9)	50 (34.7)	0.464
Current smoking, *n* (%)	48 (28.9)	31 (23.3)	0.274
**Laboratory findings**			
aPTT ratio^*^	0.93 (0.86–1.01)	0.99 (0.91–1.02)	0.003
INR^*^	1.02 (0.97–1.08)	1.02 (1.00–1.08)	0.788
Fasting plasma glucose, mg/dl^*^	110.0 (91.5–132.5)	98.0 (85.5–120.0)	0.001
**Blood pressure**			
Systolic blood pressure	163.7 ± 26.0	163.0 ± 24.8	0.824
Diastolic blood pressure^*^	90 (80–100)	90 (80–100)	0.177
**Pre-stroke antithrombotic treatment**			
Antiplatelets, *n* (%)	43 (24.6)	49 (34.0)	0.064
Anticoagulants, *n* (%)	6 (3.4)	0 (0.0)	0.001
**Post-stroke antithrombotic treatment**			
Antiplatelets, *n* (%)	138 (78.9)	109 (75.7)	0.501
of which:			
Monotherapy, *n* (%)	117 (84.8)	70 (64.2)	0.001
DAPT, *n* (%)	21 (15.2)	39 (35.8)	0.001
Anticoagulants, *n* (%)	37 (21.1)	35 (24.3)	0.501
CTA for examining extracranial vessel status†	113 (64.6)	81 (61.8)	0.610
Ipsilateral ICA stenosis ≥70%	8 (4.6)	14 (9.7)	0.071
**Stroke subtypes based on TOAST classification**			
Large arterial atherosclerosis, *n* (%)	18 (10.3)	20 (13.9)	0.778
Cardioembolism, *n* (%)	47 (26.9)	41 (28.5)	
Small vessel disease, *n* (%)	29 (16.6)	24 (16.7)	
Other determined etiology, *n* (%)	6 (3.4)	6 (4.2)	
Undetermined etiology, *n* (%)	75 (42.9)	53 (36.8)	
**Clinical characteristics**			
NIHSS score at admission^*^	3 (2–4)	1 (0.25–2)	0.001
NIHSS score at discharge^*^	0 (0–1)	0 (0–1)	0.084

IVT, intravenous thrombolysis; aPTT, activated partial thromboplastin time; INR, international normalized ratio; DAPT, dual antiplatelet therapy; CTA, CT-angiography; ICA, internal carotid artery; TOAST, Trial of ORG 10172 in Acute Stroke Treatment; NIHSS, National Institute of Health Stroke Scale.

† In all patients who did not perform CT-angiography, extracranial vessel status was investigated using an ultrasound echo-color Doppler.

Data are presented as mean and standard deviation for normally distributed continuous variables. Non-normally distributed continuous variables are displayed as median and interquartile range and are identified by an asterisk (^*^).

### Outcome measures

Table 2 summarizes the outcome measures in patients in the IVT+ and IVT– groups. We did not observe any difference regarding disability, mortality, and hemorrhagic complications between the two groups. As reported in Table 3, the use of alteplase was not significantly associated with any of the outcome measures.

**Table 2:** Outcome measures according to treatment with intravenous thrombolysis.

	**IVT + (*n* = 175)**	**IVT – (*n* = 144)**	***p***
3-month favorable outcome, *n* (%)	144 (82.3)	124 (86.1)	0.354
3-month functional independence, *n* (%)	156 (89.1)	132 (91.7)	0.449
Relevant neurological deterioration at discharge, *n* (%)	4 (2.3)	3 (2.1)	0.606
In-hospital mortality, *n* (%)	2 (1.1)	2 (1.4)	0.612
3-month mortality, *n* (%)	2 (1.1)	3 (2.1)	0.661
ICH, *n* (%)	5 (2.9)	4 (2.8)	0.620
SICH, *n* (%)	2 (1.1)	1 (0.7)	0.573

IVT, intravenous thrombolysis; ICH, intracranial hemorrhage; SICH, symptomatic intracranial hemorrhage.

**Table 3:** Multivariate logistic regression models for the outcome measures.

**Variables**	**OR**	**95% CI**	***p***
**Three-month favorable outcome**			
Age	0.96	0.93–0.98	0.003
Male sex	1.09	0.57–2.11	0.789
NIHSS score at admission	0.59	0.46–0.78	0.001
Use of alteplase	1.60	0.77-3.34	0.208
**3-month functional independence**			
Age	0.96	0.93–0.99	0.020
Male sex	1.70	0.73–3.99	0.220
NIHSS score at admission	0.58	0.42–0.79	0.001
Use of alteplase	1.59	0.65–3.89	0.307
**Relevant neurological deterioration at discharge**			
Age	1.03	0.97–1.10	0.337
Male sex	0.27	0.03–2.28	0.227
NIHSS score at admission	1.22	0.68–2.19	0.502
Use of alteplase	0.86	0.16–4.69	0.864
**In-hospital mortality**			
Age	1.05	0.96–1.15	0.319
Male sex	0.00	0.00-NA	0.996
NIHSS score at admission	2.39	1.03–5.55	0.041
Use of alteplase	0.34	0.04–2.91	0.325
**3-month mortality**			
Age	1.02	0.95–1.10	0.518
Male sex	0.00	0.00-NA	0.996
NIHSS score at admission	1.85	0.94–3.65	0.076
Use of alteplase	0.28	0.04–1.99	0.203
**Presence of ICH**			
Age	1.02	0.96–1.08	0.496
Male sex	0.48	0.09–2.41	0.374
NIHSS score at admission	1.21	0.72–2.03	0.465
Use of alteplase	0.80	0.18–3.59	0.771
**Presence of SICH**			
Age	0.98	0.90–1.07	0.655
Male sex	1.04	0.09–12.20	0.972
NIHSS score at admission	0.91	0.37–2.23	0.846
Use of alteplase	1.94	0.13–30.00	0.635

NIHSS, National Institute of Health Stroke Scale; NA, not available; ICH, intracranial hemorrhage; SICH, symptomatic intracranial hemorrhage.

## Discussion

This study suggests that the use of alteplase, although it does not increase the risk of hemorrhagic complications, is not associated with a better outcome in highly selected patients with non-disabling AIS.

The frequency of thrombolytic treatment has significantly increased over the last few years (23). This might be due to improved logistics to reduce delays in pre-hospital and in-hospital management of patients with acute stroke, enabling more patients to receive intravenous thrombolysis. However, it cannot be excluded that the increasing rate of thrombolysis is partly caused by more and more patients with minor strokes being treated. An analysis of data from the Swedish Stroke Register confirms this hypothesis, showing that, among patients receiving alteplase, the proportion with a minor stroke significantly increased from 22.1% in 2007 to 28.7% in 2010 (*p* = 0.021) (24). In our highly selected sample of patients with minor strokes, intravenous thrombolysis was frequently given, i.e., to almost 55% of the entire sample. This result might be due to our aggressive approach, which does not use a low NIHSS cutoff as an absolute treatment exclusion criterion. However, our treatment approach to mild stroke is not unique; for example, Urra et al. reported that almost 60% of their AIS patients with mild symptoms received alteplase (4). As expected, a higher NIHSS score significantly increased the odds of being treated with alteplase in our population.

Previous studies reported that about a third of AIS patients with mild symptoms were functionally dependent or would be dead if not receiving alteplase (25, 26). Differently, we observed a lower rate of poor outcomes of nearly 16%. The definition of mild stroke is probably the reason for this dissimilarity. The specific definition of “mild” has not been agreed upon universally, and there is variability in the interpretation and implementation among centers and even individual treating practitioners. The most common definition deems patients with a baseline NIHSS score of 0–5 as affected by mild stroke (26, 27). However, using the BI as a tool for measuring disability, we observed that almost one-half (51.7%) of patients with an NIHSS score of 0–5 at admission were functionally dependent, having a BI score of <80 (12). In this research, adopting only the NIHSS, we tried to identify patients with non-disabling stroke symptoms. Thus, as performed in the ARAMIS trial, we excluded patients who had substantial deficits in vision, language, neglect, and single limb and who had an altered level of consciousness. After carefully selecting our population, we observed that more than 80% of patients with mild stroke may achieve a favorable outcome. Recently, Sykora et al. (9) reported similar rates in AIS patients with an NIHSS score of 0–1 at admission.

The main finding of this observational study is that alteplase use does not improve functional outcomes in patients with non-disabling stroke. Indeed, rates of 3-month mRS 0–1 were superimposable, being 82.3% and 86.1% in the treated and untreated patients groups, respectively. Only Spokoyny et al. adopted a definition of mild stroke that was very similar to ours. Although in a smaller sample, the authors did not find any significant difference in outcomes in mild stroke patients receiving vs. not receiving intravenous thrombolysis (7). Different from individuals with non-disabling stroke, in patients with mild stroke but disabling symptoms, alteplase seems to preserve its efficacy (4–6, 28). Our previous study clearly demonstrates that alteplase is effective only when patients with mild stroke have a BI score of <80 (12). Evaluating mild stroke patients with an NIHSS score of 0–1 and those with an NIHSS score of 2–5, Sykora et al. reported that alteplase improved outcomes only in the group of subjects having higher NIHSS scores, whereas the positive effect of thrombolysis was overruled by the effects of SICH in the group with an NIHSS score of 0–1 (9). To date, the PRISMS study is the only published randomized trial that evaluated the efficacy of alteplase for the treatment of AIS with an NIHSS score of 0–5 and without clearly disabling deficits. Unfortunately, the study was terminated early because of low patient recruitment. Results of the 313 patients enrolled failed to demonstrate more favorable functional outcomes in patients treated with alteplase as compared to those receiving only aspirin (10). We are waiting for the results of the ARAMIS trial (14).

The risk of hemorrhagic complications is one of the major reasons that patients with mild stroke are excluded from treatment with alteplase. The incidence of SICH based on stroke severity was compared in a cohort study that found the risk of hemorrhage was ~2% in patients with mild stroke and 8.1% in patients with a baseline NIHSS score of more than 6 (29). Our study confirms that hemorrhagic complications are infrequent in patients with non-disabling strokes. In particular, the use of alteplase did not increase the risk of SICH in our sample, affecting only two individuals (1.1%). Similar rates of SICH were reported by Spokoyny et al. (0%) and Sykora et al. (1.4%) (7, 9).

Several limitations of this study should be considered. The retrospective design of the study may produce systematic errors and bias. Measures of outcome were obtained by physicians that were not blinded to alteplase treatment, which may have influenced their rating, causing detection bias. Since patients coming from a single center with vascular neurologists possibly used to treat or not treat non-disabling stroke symptoms with alteplase, our results cannot be generalized. As this was an observational study in which alteplase was administered as indicated by vascular neurologists, we cannot exclude that additional biases may have influenced the decision to administer or withhold alteplase. The relatively small sample size may have limited the statistical power; thus, the alteplase effectiveness in our sample could not be detected. Finally, the observed associations are no proof of causality; thus, our results should be considered hypothesis-generating.

## Conclusion

This observational study suggests that the use of alteplase, although safe, does not improve clinical outcomes in highly selected patients with non-disabling AIS. Further large interventional studies are needed to confirm these preliminary findings.

## Data availability statement

The raw data supporting the conclusions of this article will be made available by the authors, without undue reservation.

## Ethics statement

The studies involving human participants were reviewed and approved by Comitato Etico Unico Regionale (CEUR-2020-Os-173). Written informed consent for participation was not required for this study in accordance with the national legislation and the institutional requirements.

## Author contributions

Conceptualization: GM and LN. Methodology, formal analysis, data curation, writing—original draft preparation, and writing—review and editing: GM. Software, investigation, and resources: PV, MS, SP, AM, YT, GS, SL, and FJ. Validation: GG and MV. Visualization: GG. Supervision: MV. All authors contributed to the article and approved the submitted version.

## References

[1] Wardlaw JM Zoppo G Yamaguchi T Berge E. Thrombolysis for acute ischaemic stroke. Cochrane Database Syst Rev. (2003) 3:CD000213. 10.1002/14651858.CD000213 12917889

[2] Romano JG Gardener H Campo-Bustillo I Khan Y Tai S Riley N. MaRISS Investigators. Predictors of outcomes in patients with mild ischemic stroke symptoms. MaRISS Stroke. (2021) 52:1995–2004. 10.1161/STROKEAHA.120.032809 33947209 PMC9254134

[3] Huisa BN Raman R Neil W Ernstrom K Hemmen TM. Intravenous tissue plasminogen activator for patients with minor ischemic stroke. J Stroke Cerebrovasc Dis. (2012) 21:732–6. 10.1016/j.jstrokecerebrovasdis.2011.03.009 21531576 PMC3164904

[4] Urra X Ariño H Llull L Amaro S Obach V Cervera Á. The outcome of patients with mild stroke improves after treatment with systemic thrombolysis. PLoS One. (2013) 8:e59420. 10.1371/journal.pone.0059420 23527192 PMC3602063

[5] Greisenegger S Seyfang L Kiechl S Lang W Ferrari J. Austrian stroke unit registry collaborators. Thrombolysis in patients with mild stroke: results from the Austrian stroke unit. Registry Stroke. (2014) 45:765–9. 10.1161/STROKEAHA.113.003827 24481972

[6] Logallo N Kvistad CE Naess H Waje-Andreassen U Thomassen L. Mild stroke: safety and outcome in patients receiving thrombolysis. Acta Neurol Scand Suppl. (2014) 198:37–40. 10.1111/ane.12235 24588505

[7] Spokoyny I Raman R Ernstrom K Khatri P Meyer DM Hemmen TM. Defining mild stroke: outcomes analysis of treated and untreated mild stroke patients. J Stroke Cerebrovasc Dis. (2015) 24:1276–81. 10.1016/j.jstrokecerebrovasdis.2015.01.037 25906938 PMC4457618

[8] Haeberlin MI Held U Baumgartner RW Georgiadis D Valko PO. Impact of intravenous thrombolysis on functional outcome in patients with mild ischemic stroke without large vessel occlusion or rapidly improving symptoms. Int J Stroke. (2020) 15:429–37. 10.1177/1747493019874719 31514684

[9] Sykora M Krebs S Simader F Gattringer T Greisenegger S Ferrari J. Austrian stroke unit registry collaborators. Intravenous thrombolysis in stroke with admission NIHSS score 0 or 1. Int J Stroke. (2022) 17:109–19. 10.1177/1747493021991969 33568019

[10] Khatri P Kleindorfer DO Devlin T Sawyer RN Jr Starr M Mejilla J. PRISMS Investigators. Effect of alteplase vs aspirin on functional outcome for patients with acute ischemic stroke and minor nondisabling neurologic deficits: the PRISMS randomized clinical trial. JAMA. (2018) 320:156–66. 10.1001/jama.2018.8496 29998337 PMC6583516

[11] Ferrari J Reynolds A Knoflach M Sykora M. Acute ischemic stroke with mild symptoms-To thrombolyse or not to thrombolyse? Front Neurol. (2021) 12:760813. 10.3389/fneur.2021.760813 34867745 PMC8637329

[12] Merlino G Smeralda C Lorenzut S Gigli GL Surcinelli A Valente M. To treat or not to treat: Importance of functional dependence in deciding intravenous thrombolysis of “mild stroke” patients. J Clin Med. (2020) 9:768. 10.3390/jcm9030768 32178336 PMC7141285

[13] Schwartz JK Capo-Lugo CE Akinwuntan AE Roberts P Krishnan S Belagaje SR. Classification of mild stroke: a mapping review. PM R. (2019) 11:996–1003. 10.1002/pmrj.12142 30746896

[14] Wang XH Tao L Zhou ZH Li XQ Chen HS. Antiplatelet vs. R-tPA for acute mild ischemic stroke: A prospective, random, and open label multi-center study. Int J Stroke. (2019) 14:658–63. 10.1177/1747493019832998 30907301

[15] Jauch EC Saver JL Adams HP Jr Bruno A Connors JJ Demaerschalk BM. American Heart Association Stroke Council. Council on Cardiovascular Nursing Council on Peripheral Vascular Disease Council on Clinical Cardiology Guidelines for the early management of patients with acute ischemic stroke: a guideline for healthcare professionals from the American Heart Association. Stroke. (2013) 44:870–947. 10.1161/STR.0b013e318284056a 23370205

[16] Powers WJ Rabinstein AA Ackerson T Adeoye OM Bambakidis NC Becker K. American Heart Association Stroke Council. 2018 Guidelines for the Early Management of Patients With Acute Ischemic Stroke: A Guideline for Healthcare Professionals From the American Heart Association/American Stroke Association. Stroke. (2018) 49:e46-e110. 10.1161/STR.0000000000000158 29367334

[17] Powers WJ Rabinstein AA Ackerson T Adeoye OM Bambakidis NC Becker K. Guidelines for the early management of patients with acute ischemic stroke: 2019 update to the 2018 guidelines for the early management of acute ischemic stroke: a guideline for healthcare professionals from the American Heart Association/American Stroke Association. Stroke. (2019) 50:e344–418. 10.1161/STR.0000000000000211 31662037

[18] Heidbuchel H Verhamme P Alings M Antz M Hacke W Oldgren J. EHRA practical guide on the use of new oral anticoagulants in patients with non-valvular atrial fibrillation: executive summary. Eur Heart J. (2013) 34:2094–106. 10.1093/eurheartj/eht134 23625209

[19] North American Symptomatic Carotid Endarterectomy Trial Collaborators Barnett HJM Taylor DW Haynes RB Sackett DL Peerless SJ. Beneficial effect of carotid endarterectomy in symptomatic patients with high-grade carotid stenosis. N Engl J Med. (1991) 325:445–53. 10.1056/NEJM199108153250701 1852179

[20] Adams HP Jr Bendixen BH Kappelle LJ Biller J Love BB Gordon DL. Definitions for use in a multicenter clinical trial. TOAST. Trial of Org 10172 in Acute Stroke Treatment. Stroke. (1993) 24:35–41. 10.1161/01.str.24.1.35 7678184

[21] Hacke W Kaste M Fieschi C Toni D Lesaffre E von Kummer R. Intravenous thrombolysis with recombinant tissue plasminogen activator for acute hemispheric stroke. The European cooperative acute stroke study (ECASS). JAMA. (1995) 274:1017–25. 10.1001/jama.1995.03530130023023 7563451

[22] Hacke W Kaste M Bluhmki E Brozman M Dávalos A Guidetti D. Thrombolysis with alteplase 3 to 45 hours after acute ischemic stroke. N Engl J Med. (2008) 359:1317–29. 10.1056/NEJMoa0804656 18815396

[23] Olavarría VV Hoffmeister L Vidal C Brunser AM Hoppe A Lavados PM. Temporal trends of intravenous thrombolysis utilization in acute ischemic stroke in a prospective cohort from 1998 to 2019: modeling based on joinpoint regression. Front Neurol. (2022) 13:851498. 10.3389/fneur.2022.851498 35463124 PMC9028765

[24] Stecksén A Asplund K Appelros P Glader EL Norrving B Eriksson M. Riks-Stroke Collaboration. Thrombolytic therapy rates and stroke severity: an analysis of data from the Swedish stroke register. (Riks-Stroke) 2007-2010. Stroke. (2012) 43:536–8. 10.1161/STROKEAHA.111.630590 21980204

[25] Khatri P Conaway MR Johnston KC. Acute stroke accurate prediction study. (ASAP) Investigators Ninety-day outcome rates of a prospective cohort of consecutive patients with mild ischemic stroke. Stroke. (2012) 43:560–2. 10.1161/STROKEAHA.110.593897 22052513 PMC3426999

[26] Fischer U Baumgartner A Arnold M Nedeltchev K Gralla J De Marchis GM. What is a minor stroke? Stroke. (2010) 41:661–6. 10.1161/STROKEAHA.109.572883 20185781

[27] Khatri P Kleindorfer DO Yeatts SD Saver JL Levine SR Lyden PD. Strokes with minor symptoms: an exploratory analysis of the national institute of neurological disorders and stroke recombinant tissue plasminogen activator trials. Stroke. (2010) 41:2581–6. 10.1161/STROKEAHA.110.593632 20814000 PMC2964419

[28] You S Saxena A Wang X Tan W Han Q Cao Y. Efficacy and safety of intravenous recombinant tissue plasminogen activator in mild ischaemic stroke: a meta-analysis. Stroke Vasc Neurol. (2018) 3:22–7. 10.1136/svn-2017-000106 29600004 PMC5870640

[29] Zhao D Liu J Wang W Zeng Z Cheng J Liu J. Epidemiological transition of stroke in China: 21-year observational study from the Sino-MONICA-Beijing Project. Stroke. (2008) 39:1668–74. 10.1161/STROKEAHA.107.502807 18309149

